# Coordinating external assistance for UHC: Pakistan’s early experience of the SDG3 GAP

**DOI:** 10.1093/heapol/czad105

**Published:** 2023-11-18

**Authors:** Faraz Khalid, Sabeen Afzal, Ali Shirazi, Isadora Quick, Awad Mataria

**Affiliations:** Universal Health Coverage/Health Systems, World Health Organization , Regional Office for the Eastern Mediterranean, Monazamet El Seha El Alamia Street Extension of Abdel Razak El Sanhouri Street, Nasr City, Cairo 7608, Egypt; Health Wing, Ministry of National Health Services, Regulation and Coordination, Pakistan Secretariat, Islamabad 44020, Pakistan; Health Systems Cluster, World Health Organization, Pakistan Country Office, Park Road, Chak Shahzad, Islamabad 45500, Pakistan; Secretariat of the Global Action Plan for Healthy Lives and Well-being for All, Office of the Director-General, World Health Organization Headquarters, Avenue Appia 20, Geneva 1211, Switzerland; Universal Health Coverage/Health Systems, World Health Organization , Regional Office for the Eastern Mediterranean, Monazamet El Seha El Alamia Street Extension of Abdel Razak El Sanhouri Street, Nasr City, Cairo 7608, Egypt

**Keywords:** Donor coordination, external assistance for health, primary health care, sustainable health financing, sustainable development goals, universal health coverage

## Abstract

Pakistan is a lower middle-income setting. External assistance for health and development partners play an important role in health sector development. The federal system and health care delivery as a devolved provincial subject warrant regular and effective coordination among federating units and partners. Pakistan was selected as a priority country in 2019 for the implementation of the Global Action Plan for Healthy Lives and Well-being for All (SDG3 GAP). Given the ongoing universal health coverage (UHC) reforms, the country prioritized two SDG3 GAP accelerators, related to primary health care (PHC) and sustainable financing for health (SFH). Eight partner agencies representing PHC and SFH accelerators jointly planned and conducted a ‘PHC for UHC mission’ to Pakistan in 2021. This mission paved the way for setting up an SDG3 GAP Coordination Committee and a ‘PHC Service Delivery and Financing working group’, which have been regularly coming together through in-person and virtual meetings; the latter was relatively uncommon previously and this new way of working provided a chance to build rapport, share workplans, identify duplications in technical assistance and jointly gauge governments’ priorities. This has shifted the focus of reforms deliberations from ‘what’ to ‘how’, enabling joint strategic planning and implementation. The joint work by SDG3 GAP partners with the Ministry of National Health Services Regulation & Coordination linked discussions on health financing and service delivery reforms for the first time, and has contributed to advocacy, analysis, strategic policy dialogue, institutional strengthening and implementation of UHC reforms, with a focus on PHC. Even though joint work by SDG3 GAP partners undoubtedly shows the potential for better alignment and collaboration, translation of the commitment to better collaboration into concrete impact has been the result of committed and engaged staff members, rather than institutionalized change, which will require strong incentives for collaboration.

Key messages

- Momentum for universal health coverage (UHC) in Pakistan has been galvanized by closer collaboration among development partners through the SDG3 GAP platform and strong leadership by the Ministry of National Health Services Regulation & Coordination (NHSR&C)
- Partners’ coordination with federal and provincial governments on health financing and service delivery reforms has improved and has enabled the achievement of several important milestones, including consensus towards the development of a national health financing framework, provincial adaptation of the Essential Package of Health Services (EPHS) and launch of a National Health Support Program for implementing EPHS at the sub-national level.
- Joint work by SDG3 GAP partners in Pakistan has undoubtedly shown potential for better alignment and collaboration. Translation of the commitment to better collaboration into concrete impact has been the result of committed and engaged staff members, rather than institutionalized change, which will require strong incentives for collaboration.
- Unprecedented floods, the recent political and economic situation and limited capacity for partner coordination at the provincial level have been the major constraints for extending the scope of the ongoing collaborative agenda. The report provides useful lessons learned on what closer collaboration at country level entails and can achieve.

## Introduction

Pakistan is a lower middle-income setting and is the fifth most populous country in the world (Government of Pakistan, Finance Division, 2022). Though health indicators have improved over the last 70 years, progress has been slow in comparison to countries with a similar socio-economic profile (Nishtar *et al.*, 2013; Hafeez *et al.*, 2023). The last two decades saw several supply and demand side reforms in the health sector, but primary health care (PHC) has not received adequate attention, resulting in an inverted health care delivery pyramid and a disease-focused approach, which have impeded progress towards universal health coverage (UHC) (World Health Organization and Alliance for Health Policy and Systems Research, 2017). Pakistan’s chronic under-funding of the health system is one of the determinants of poor health indicators. Current health expenditure (CHE) has been marginally increasing every year and has gone from USD 27.91 per person in 2009–10 to USD 40.7 per person in 2019–20, while General Government Health Expenditure from domestic sources (GGHE-D) represented only 1.2% of its GDP in 2020–21. Low government spending has been associated with high out-of-pocket payments, which have been consistently >50% in the last 15 years and stood at 52.9% in 2019–20 (Government of Pakistan, Pakistan Bureau of Statistics, 2022; World Health Oganization, 2023).

In 2016, Pakistan finalized its National Health Vision 2016–25, which acknowledges the significance of PHC as the foundation of health system reforms (Ministry of National Health Services Regulation and Coordination, 2016). Through their external assistance for health, development partners have been playing an important role in supporting the country to achieve its Vision’s objectives, and several development partners have large portfolios in Pakistan (Gavi, the Vaccine Alliance, 2023; The Global Fund, 2023; UNICEF, 2023; World Bank, 2023; World Health Organizatoin, 2023). Given the complex federal system and devolved provincial authority in health, effective coordination among federating units and development partners is critical. Several development and humanitarian partners’ fora for coordination have been active in Pakistan but have mostly focused on specific disease programmes. There has been a need to accelerate and harmonize the efforts for PHC strengthening and sustainable financing for health by using common tools, instruments and approaches.

In 2019, Pakistan was selected as a priority country for the implementation of the Global Action Plan for Healthy Lives and Well-being for All (SDG3 GAP), which is a self-commitment of 13 multilateral agencies^1^ active in health to better support countries to achieve the health-related Sustainable Development Goals (SDGs) through closer and improved collaboration. It provides a platform for joint action at country level around thematic areas identified by the signatory agencies as accelerators, with the aim of aligning ways of working and reducing inefficiencies.^2^ Given the ongoing UHC reforms, including the development and implementation of the UHC Benefit Package/Essential Package of Health Services (EPHS) and the prioritization of PHC in provincial health plans, the country prioritized two SDG3 GAP accelerators of PHC and sustainable financing for health (SFH) (World Health Organization, 2021).

Through this report, the co-authors share their joint experience on how regular activities of the SDG3 GAP partners have been supporting UHC reforms in the country, and highlight the implementation process, key areas of joint progress, enablers, barriers and main challenges encountered in strengthening the collaboration.

## Implementation and achievements (keys areas of joint progress)

Harnessing the momentum for UHC reforms, eight SDG3 GAP partner agencies, collaborating mostly on the PHC and SFH accelerators, jointly planned and conducted a ‘PHC for UHC mission’ to Pakistan in March 2021. This led to a high-level national ‘PHC4UHC’ Forum (a 1-day meeting), where representatives of federal and provincial governments and SDG3 GAP partners signed a joint statement in support of enhancing PHC towards UHC in Pakistan (World Health Organization, Regional Office for the Eastern Mediterranean, 2021).

Further, the mission paved the way for the establishment of an SDG3 GAP Coordination Committee and a ‘PHC Service Delivery and Financing working group’ in the same year.

The SDG3 GAP Coordination Committee brings together country representatives from Gavi, the Global Financing Facility (GFF), the Global Fund, Joint United Nations Programme on HIV/AIDS (UNAIDS), United Nations Population Fund (UNFPA), United Nations Children’s Fund (UNICEF), World Health Organization (WHO), World Bank and the Ministry of National Health Services Regulation & Coordination (Ministry of NHSR&C); the committee meets in-person periodically and provides strategic oversight for joint activities undertaken by the ‘PHC Service Delivery and Financing working group’, which is represented by the partners’ technical focal points (at the country, regional and global levels). This working group is co-led by WHO, World Bank and the Global Fund and meets virtually every few months. The group meetings were initially attended by SDG3 GAP partners only, but as needed and based upon their interest, bilateral donors/partners have been invited to the meetings. Virtual meeting platforms were initially used due to COVID-19 pandemic and related restrictions on mobility and travel, and later, finding the virtual modality convenient for convening partner representatives, almost all the meetings have been convened virtually.

The Ministry of NHSR&C has been playing a pivotal role in setting the agenda for UHC reforms through evidence generation and strategic dialogue at the federal and provincial levels, and has been strongly demanding closer collaboration, which has led to a more regular engagement of the development partners’ country representatives with the Ministry. These interactions have informed the deliberations of the SDG3 GAP Coordination Committee and the ‘PHC Service Delivery and Financing working group’ and have enabled participation of federal and provincial departments of health representatives in working group meetings.

Through the SDG3 GAP, closer partner collaboration and joint work on PHC and SFH with the Ministry of NHSR&C have resulted in linking discussions on health financing and service delivery reforms for the first time in the country, and contributed to advocacy, analysis, strategic policy dialogue, institutional strengthening and implementation of UHC reforms, with a focus on PHC.

In support of a sustainably financed PHC for UHC, partners have been supporting federal and provincial governments with several analyses. Health financing assessments by the World Bank and WHO offer an empirical foundation and qualitative review of the progress against the desirable health financing attributes and are contributing to the development of the national health financing framework. The GFF and the World Bank are conducting resource mapping and expenditure tracking and an in-depth analysis of public financial management at the district level; these analyses will address the data gaps in PHC financing at the district level. GFF, UNICEF and World Bank have jointly developed a UHC investment case based on six priority reforms and coverage targets of critical PHC-based EPHS interventions. WHO, in collaboration with Gavi and the Global Fund, has conducted a cross-programmatic efficiency analysis of selected vertical programmes to identify inefficiencies across these and to address the bottlenecks through a health system strengthening lens.

SDG3 GAP partners have been engaged in thorough consultations and strategic dialogue with federal and provincial governments on the provincial adaptation of EPHS, and consequently all the provinces except one have localized the EPHS within their plans. Joint work has also led to partners aligning their upcoming portfolios with the governments’ priority for implementing the EPHS at provincial levels: (1) World Bank, GFF, Global Fund, Gavi and Bill Melinda Gates Foundation are jointly supporting a National Health Support Program, which includes the Disbursement-Linked Indicator mechanism to promote integrated programming with existing programmes and promote equitable access in remote and marginalized areas; and (2) WHO has piloted implementation of EPHS using a PHC-oriented Model of Care approach in selected districts, and its experience would feed into the overall implementation and scale-up of EPHS across the country.

Through the ‘PHC Service Delivery and Financing working group’ and ‘SDG3 GAP Coordination Committee’, the focus of reforms deliberations has shifted from ‘what’ to ‘how’ and has enabled joint strategic planning and implementation. Additionally, SDG3 GAP partners have supported the governments in keeping equity at the heart of UHC reforms; the equity focus has been built into the review of the Federal Directorate of Immunization (FDI) and the development of the Lady Health Worker National Strategic Plan (2022–2026). Similarly, the focus on the equity agenda is helping the country make progress in reaching ‘zero dose’ communities in districts with the lowest coverage.

Several institutional strengthening activities are underway to support the National Health Accounts and controller general of accounts, which will consequently feed into public financial management reforms. Further, SDG3 GAP partners are supporting the Ministry of NHSR&C in strengthening its capacity in generating quality evidence and its timely dissemination to relevant stakeholders.

While making joint progress, in 2021, the Ministry of NHSR&C and SDG3 GAP partners volunteered to pilot the questionnaire of the SDG3 GAP monitoring framework^3^ assessing what worked and what didn’t under the SDG3 GAP coordination platform; this questionnaire was administered by the SDG3 GAP secretariat (World Health Organization, 2023a).

## Enablers

The key enablers for joint progress have been as follows.

- The leadership provided by the Ministry of NHSR&C has played a critical role in identifying evidence gaps, windows of opportunity at provincial levels in translating the evidence into policy and practice, and engaging partners for meeting country needs. Further, its leadership has been essential in demanding and steering closer partner collaboration, as was well reflected in the Ministry’s response to the SDG3 GAP questionnaire for national governments.
- As collaboration requires up-front resources, dedicated individuals in both the Ministry of NHSR&C and partner organizations have been integral to developing and implementing joint actions. These individuals considered SDG3 GAP a timely opportunity to revitalize partners’ coordination and align partners’ portfolios/investments with the UHC momentum in the country.
- The focus on ‘equity’ brought partners and governments together on aligning technical and political priorities with on-the-ground needs.
- The virtual working environment as a consequence of the COVID-19 pandemic enabled partner organizations’ representatives from different tiers (global, regional and country) to frequently come together, which was uncommon previously, and this new way of working helped partners to build rapport among themselves, share workplans, identify duplications in technical assistance and jointly gauge governments’ priorities.

## Challenges and barriers

The process has not been free from challenges and the main barriers to the effective functioning of the SDG3 GAP platform are as follows.

- Institutional ways of working of partner agencies are slow to change and it has been taking some time to shift the focus from the prevailing culture of working in silos, with a focus on disease programmes within government departments and partners, to working together for integrated health services delivery through a PHC approach.
- As reflected in the 2023 SDG3 GAP progress report, (World Health Organization, 2023b) which provides an honest self-assessment of what worked and what did not since the SDG3 GAP was launched in 2019, the translation of the self-commitment by partners to improved collaboration at country level can only achieve so much. Without strong incentives for collaboration, it remains difficult to change the ways of working of organizations and bring about transformational change.
- Unprecedented floods in Pakistan in 2022, which affected one-third of the country (United Nations Office for the Coordination of Humanitarian Affairs, 2022), diverted attention and resources from development towards urgent humanitarian needs, and partners had to repurpose their plans and resources.
- Post-devolution, provinces are autonomous in making their sectoral decisions, and though partners’ coordination platforms do exist at the provincial level, their capacity is limited. Hence, SDG3 GAP partners’ coordinated engagement with provincial governments and health departments is limited at this stage.

## Conclusions

By seizing the opportunity presented by the SDG3 GAP platform, development partners have engaged with the Ministry of NHSR&C on UHC reforms in a more coordinated manner, and the Ministry has played a critical role in steering technical analyses and strategic dialogues and engaging partners as per their comparative advantages. This has paved the way for several important milestones, including reaching consensus on the development of a national health financing framework, the provincial adaptation of EPHS and launching the National Health Support Program for implementing EPHS at the sub-national level.

Closer and more effective collaboration among development partners played an instrumental role in achieving these milestones. However, even though joint work by SDG3 GAP partners undoubtedly showed potential for better alignment and collaboration, translation of the commitment to better collaboration into a concrete impact has been the result of committed and engaged staff members, rather than institutionalized change. To sustainably change ways of working of partner agencies, incentives in support of collaboration need to be strengthened in three key areas: (1) increasing the accountability of development partners to national governments and their priorities and plans; (2) strengthening governance direction provided by the governing bodies of the partner agencies, promoting closer collaboration; and (3) making funding for collaboration available. Further, there is a need for operational research on the relationship and power dynamics between and within SDG3 GAP partners and the Ministry of NHSR&C, which will offer useful lessons for the next set of activities entailing coordination and alignment.

This report, through experience sharing, provides useful insights into the coordination required at national and provincial levels in Pakistan, analyses key enablers and challenges, and provides lessons that may be relevant for development partners and governments jointly implementing UHC reforms in similar contexts.

## Endnotes

1. The signatories to the SDG3 GAP are: Gavi, the Vaccine Alliance; Global Financing Facility for Women, Children and Adolescents (GFF); International Labour Organization (ILO); The Global Fund to Fight AIDS, TB and Malaria; Joint United Nations Programme on HIV/AIDS (UNAIDS); United Nations Development Programme (UNDP); United Nations Population Fund (UNFPA); United Nations Children’s Fund (UNICEF); Unitaid; United Nations Entity for Gender Equality and the Empowerment of Women (UN Women); World Bank Group; World Food Programme (WFP); and World Health Organization (WHO).

2. The seven SDG3 GAP accelerator themes are: primary health care; sustainable financing for health; determinants of health; civil society and community engagement; innovative programming in fragile and vulnerable settings and programming in the context of disease outbreaks; research and development/innovation and access; and data and digital health. The commitment to gender equality cuts across accelerator themes.

3. The questionnaire allows governments to share their perspective on how well development partners are aligning to national priorities and plans and how well they are collaborating with each other. It also provides the government with the opportunity to make suggestions for improvement. Notwithstanding its limitations, such exercise strengthens the country’s ownership of the collaboration agenda and creates a demand for better coordination. It also provides a basis for development partners to discuss their joint work and reflect on how to improve it, and thus increases their accountability to governments.

## Reflexivity statement

F.K. (male) was working with WHO’s regional office for the Eastern Mediterranean in Egypt at the time of submitting the abstract in 2022 and had supported SDG3 GAP activities in Pakistan from the regional office; he is currently Research & Innovation Officer at Special Program on Primary Health Care, WHO Headquarters in Switzerland. S.A. (female) is Deputy Director Programs/Health System at the Ministry of National Health Services, Regulation and Coordination, Islamabad, Pakistan, and has been the focal point for all the coordination between SDG3 GAP partners and Ministry. A.S. (male) is Team Lead Health System Development, at WHO Country Office, Islamabad, Pakistan, and has been coordinating joint activities on behalf of WHO at the country level with SDG3 GAP partners. I.Q. (female) is Technical Officer at the SDG3 GAP Secretariat in WHO Headquarters and is part of the team that has recently reviewed responses from governments and SDG3 GAP partners on ‘What worked? What didn’t? What’s next? 2023 progress report on the Global Action Plan for Healthy Lives and Well-being for All’. A.M. (male) is Director, Universal Health Coverage/Health Systems at WHO Regional Office for Eastern Mediterranean, Cairo, Egypt, and had led the joint ‘PHC for UHC’ mission to Pakistan.
